# First Characterization of Bacterial Pathogen, *Vibrio* *alginolyticus*, for *Porites* *andrewsi* White Syndrome in the South China Sea

**DOI:** 10.1371/journal.pone.0075425

**Published:** 2013-09-24

**Authors:** Xie Zhenyu, Ke Shaowen, Hu Chaoqun, Zhu Zhixiong, Wang Shifeng, Zhou Yongcan

**Affiliations:** 1 Hainan Key Laboratory for Sustainable Utilization of Tropical Bioresource, Hainan University, Haikou, P R China; 2 Hainan Provincial Key Laboratory for Tropical Hydrobiology and Biotechnology, Hainan University, Haikou, P R China; 3 CAS Key Laboratory of Marine Bio-resources Sustainable Utilization, South China Sea institute of Oceanology, Chinese Academy of Sciences, Guangzhou, P R China; University of Connecticut, United States of America

## Abstract

**Background:**

White syndrome, a term for scleractinian coral disease with progressive tissue loss, is known to cause depressed growth and increased morality of coral reefs in the major oceans around the world, and the occurrence of this disease has been frequently reported in the past few decades. Investigations during April to September in both 2010 and 2011 identified widespread *Porites* *andrewsi* White syndrome (PAWS) in Xisha Archipelago, South China Sea. However, the causes and etiology of PAWS have been unknown.

**Methodology/Principal Findings:**

A transmission experiment was performed on *P* *. andrewsi* in the Qilianyu Subgroup (QLY). The results showed that there was a significant (P ≤ 0.05) difference between test and control groups after 28 days if the invalid replicates were excluded. Rates of tissue loss ranged from 0.90-10.76 cm^2^ d^-1^ with a mean of 5.40 ± 3.34 cm^2^ d^-1^ (mean ± SD). Bacterial strains were isolated from the PAWS corals at the disease outbreak sites in QLY of the Xisha Archipelago, South China Sea, and included in laboratory-based infection trials to satisfy Koch’s postulates for establishing causality. Following exposure to bacterial concentrations of 10^5^ cells mL^-1^, the infected colonies exhibited similar signs to those observed in the field. Using phylogenetic 16S rRNA gene analysis, classical phenotypic trait comparison, Biolog automatic identification system, MALDI-TOF mass spectrometry and MALDI Biotyper method, two pathogenic strains were identified as *Vibrio* *alginolyticus* *.*

**Conclusion/Significance:**

This is the first report of *V* *. alginolyticus* as a pathogenic agent of PAWS in the South China Sea. Our results point out an urgent need to develop sensitive detection methods for *V* *. alginolyticus* virulence strains and robust diagnostics for coral disease caused by this and *Vibrio* pathogenic bacterium in the South China Sea.

## Introduction

Coral disease, caused by different microbes, is a progressing threat to the reefs in all three major oceans around the world [1,2]. The most serious diseases, tissue loss disease termed white syndromes (WS), white band and white pox, were believed to have been principal factors in the decline of the once dominant corals in the Caribbean and in the Indo-Pacific [3,4]. Like coral bleaching, in most cases, WS also causes the loss of all or some of the symbiotic algae and photosynthetic pigments in coral animals, with the white calcium carbonate skeleton becoming visible. In the South China Sea, many diseases, such as WS, white spot disease, coral black disease, yellow inflammatory-like syndrome, pink disease and brown band disease, occurred recently in some stony corals, and the coverage of live coral has declined by more than 30% over the past few decades [5-8]. However, despite the one ciliate documented to link to brown band disease, little information is available regarding the species identities of the microbial pathogens of coral diseases in this area.

**Figure 1:**
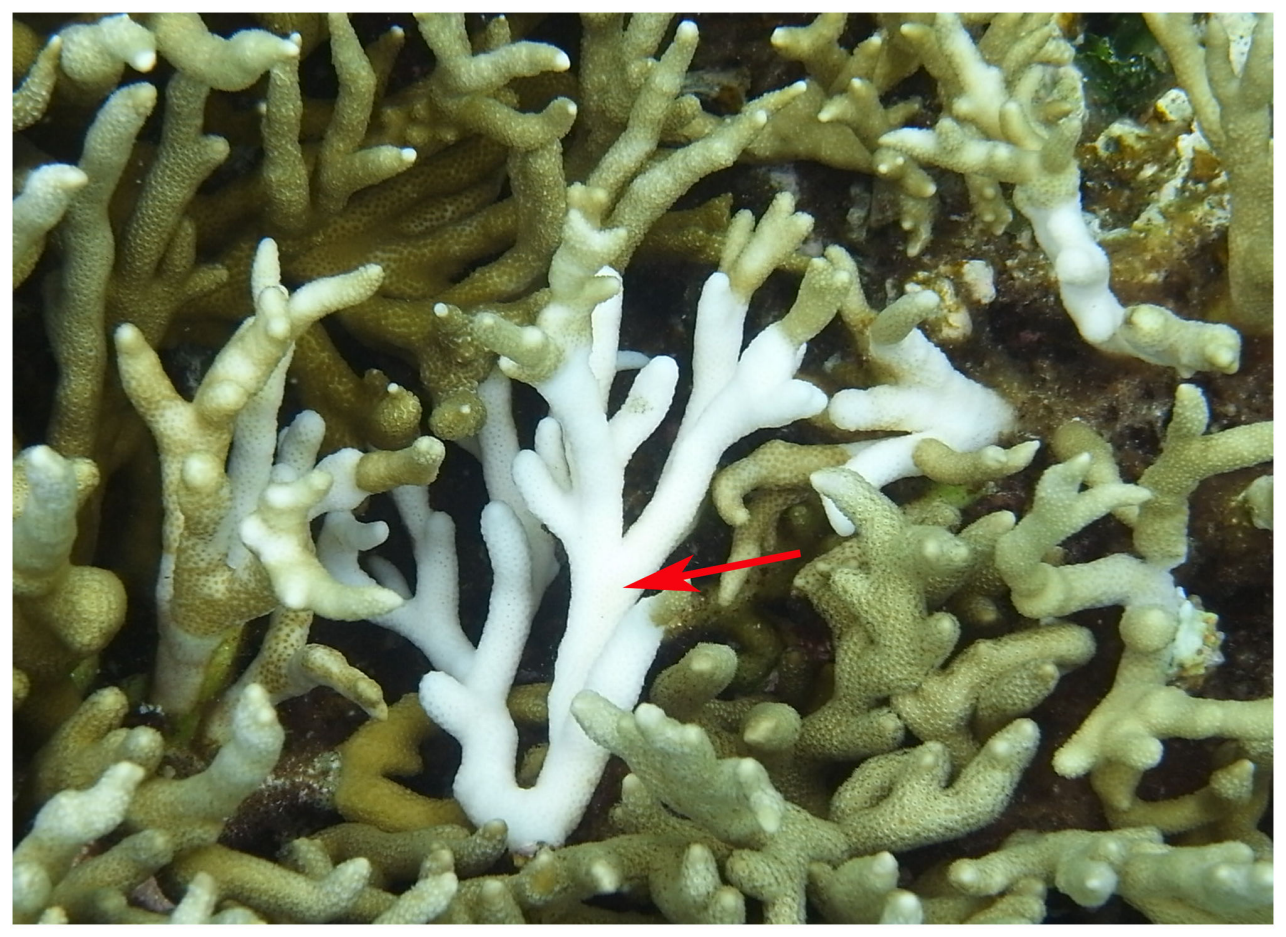
White syndrome of *Porites* *andrewsi* . Colony showing typical signs of PAWS at Nanshazhou Lagoon. The central colony (arrowed) exhibits severe tissue loss, where nearly the whole skeleton was denuded very recently (bright white). The size of the tissue loss area of the central individual was greater than those of surrounding individuals.

The Qilianyu Subgroup (QLY), named after more than seven linked islets or sandbanks, is located to the north of the Xisha Archipelago, South China Sea. Many lagoons are distributed among the islets in QLY. *Porites* *andrewsi* has been the dominant stony coral at a depth of 1-3 m in most lagoons in this sea area since 2006. However, widespread *P* *. andrewsi* White Syndrome (PAWS) in QLY was observed during the investigation period of this study, April to September in 2010 and 2011. Individuals were bright white when tissue newly lost and dead. A newly dead coral became polluted and the color became a little darker (dull white) within 4-7 d. Some algae reappeared on the surface of the dead individual after 10 d. Small white areas comprising no more than 15 individuals on average were frequently discovered. Interestingly, bright white individuals were often present around the dull white individuals, and the size of white areas of the central individuals was typically larger than that of surrounding individuals (Figure 1). These features suggest that the PAWS might be an infectious epizootic disease.

Over the past three decades, many novel coral diseases have emerged around the world [9,10]. However, few of these diseases have been characterized beyond a visual description of symptoms, and even fewer have been associated with a pathogen that satisfies Koch’s postulates [10]. *Vibrio* is a dominant species in seawater ecosystems and the main bacterial causative agent of many diseases of marine animals including corals [11-16]. Up until now, of the eight identified coral bacterial pathogens, six belong to *Vibrio* ( *V* *. alginolyticus* , *V* *. shiloi* , *V* *. coralliilyticus* *, V. natriegens*, *V. parahaemolyticus* and *V. harveyi*) [14,17,18]. Among the numerous inoculation techniques, immersion has been proven to be the most effective method for the inoculation of coral pathogens [14,16,19,20].

In this study, two types of immersion methods were employed: one-step immersion and two-step immersion. In one-step immersion, corals were immersed in inoculum during the whole experimental period. In two-step immersion, corals were immersed in inoculum only at the beginning, and transferred into seawater with no inoculum. To identify the coral pathogen according to Koch’s postulates, a highly selective enrichment medium for marine *Vibrio* spp., thiosulphate citrate bile sucrose (TCBS) agar [21], was used to isolate test strains from diseased *P* *. andrewsi* exhibited tissue loss. Furthermore, to rapidly isolate highly virulent strains, two-step immersion was selected for the inoculating mixed bacterial suspension, and one-step immersion was utilized for inoculating single bacterial suspensions. Through field and laboratory examination, two strains of *V* *. alginolyticus* were identified as the causative agents of PAWS in the Xisha Archipelago, South China Sea.

## Materials and Methods

### Transmission experiment in the field

**Figure 2:**
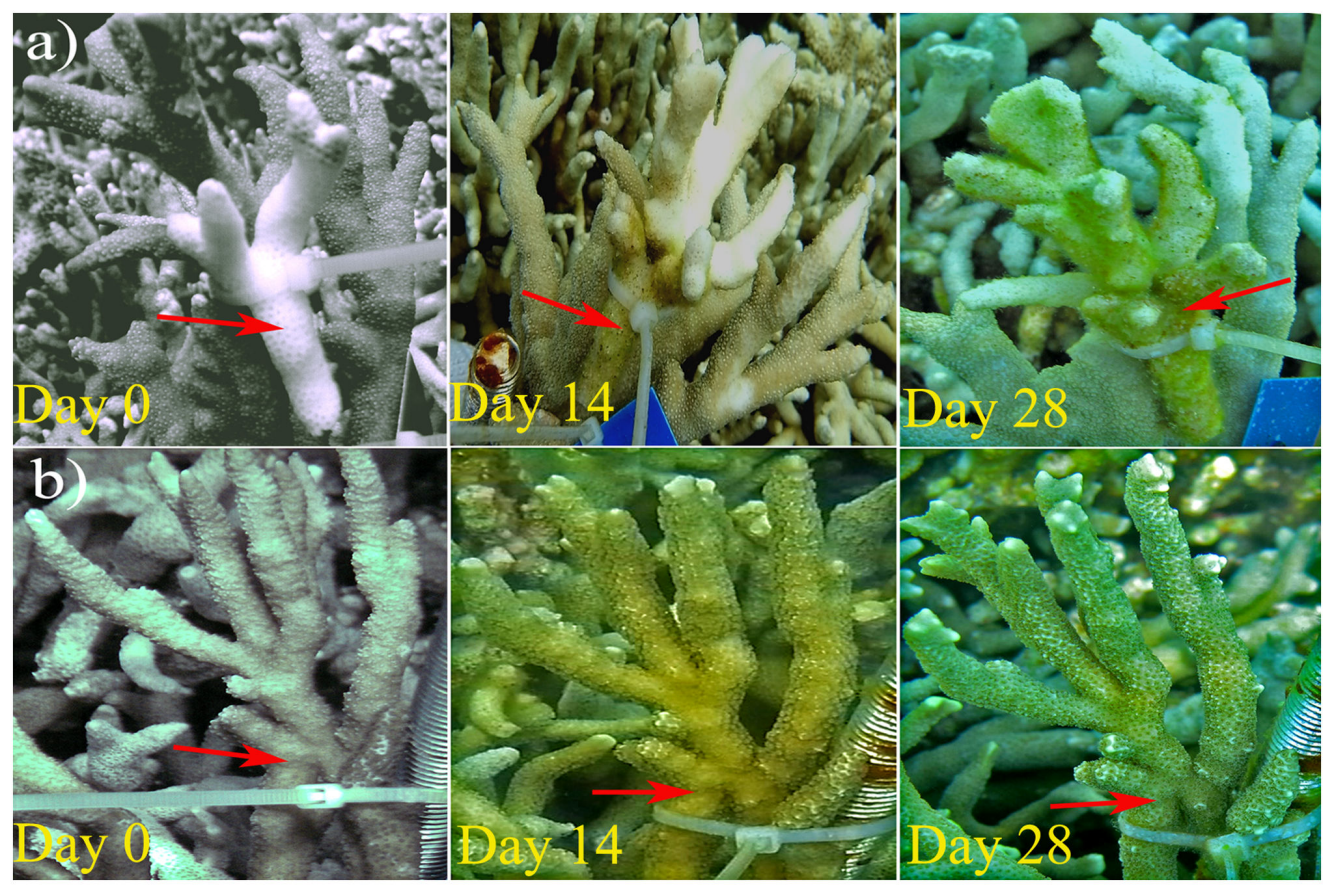
Transmission experiment of PAWS in the field. The donors are indicated by the red arrows. At initial setup (Day 0) of the treatments (a), healthy fragments in direct contact with diseased *P* *. andrewsi* fragments were healthy; no replicates had yet responded five days later; 14 d later, the *P* *. andrewsi* fragments in direct contact with diseased *P* *. andrewsi* and nearby fragments were infected; 28 d later, additional *P* *. andrewsi* fragments were also diseased. However, the control (b) *P* *. andrewsi* fragments in direct contact with healthy *P* *. andrewsi* were healthy during the entire experimental period.

This experiment was conducted at QLY (16°55.546′ N, 112°20.808′ E) at a depth of one to two meters, where naturally occurring diseased *P* *. andrewsi* , with acute signs of tissue losses often resulting in total colony mortality, were distributed sporadically among the healthy corals. Twenty-four apparently healthy fragments (nubbins) of *P* *. andrewsi* (length 20-30 cm) were collected as test receptors. Meanwhile, 16 apparently bright white 5-8 cm colonies were collected as disease donors and 8 entirely healthy 5-8 cm colonies as healthy control donors. To help sustain and attach the test corals, twenty-four one meter stainless steel bars arranged into two lines (two trials) were hammered into the reef area (Figure 2). Both trials were positioned perpendicular to the current to avoid cross-contamination between replicates through the water. The bars in each trial were two to four meters apart, and the two trials were separated by nearly 30 meters. Using cable ties, a test receptor coral and a blue plastic tag were attached to each bar. Of the 12 receptor corals in each trial, eight were paired in parallel with diseased donors (test) bundled by cable ties and four with healthy control donors (control). Thus, in each trial, there were eight test replicates and four control replicates. Latex gloves were worn and changed after the handling of each replicate to minimize cross-contamination. All 16 test replicates and eight control replicates were checked for a response (defined as the observation of tissue loss) at 5, 14 and 28 days after the trials were established. Differences in the frequency of positive (disease) responses between treatments (healthy control and disease-exposed) were analyzed using Fisher’s exact 1-tailed test (≤ 0.05; StatSoft 2001). For the replicates that responded, the response time was calculated from the day of setup (Day 0) to the day that WS was first observed. Tissue loss area was estimated from scaled photographs of fragments exhibiting a disease response. Image analysis software (Image Tool Version 3.0, UTHSCSA) was used to measure the length and diameter of the diseased areas, and the surface area was calculated based on the formula for an open-ended cylinder. The rate of tissue loss (cm^2^ d^-1^) was calculated based on the number of days between photographs. Permits for this study were issued by the Department of Ocean and Fisheries of Xisha Committee, China.

### Bacterial isolation

Three diseased nubbins (diseased samples) and three healthy nubbins (healthy samples) of *P* *. andrewsi* were randomly collected from different sites in QLY for bacterial isolation. Individual coral nubbins were placed immediately into sterile zip-lock plastic bags under water and stored on ice until the laboratory analysis. In the laboratory, samples were triply rinsed with sterile seawater (SSW). Then, 3 cm^2^ of tissue and skeleton were scraped from each sample with a steel chisel. To establish the optimal number of colonies on a culture plate, samples were plated at different dilutions (10°, 10^-1^, 10^-2^, 10^-3^, 10^-4^ in SSW). Two hundred microliters of each dilution was spread on TCBS agar (Difco, Detroit, MI, USA) plates and then incubated at 30 °C for 48 h. Upon establishment of the optimal dilution, the most abundant colonies on the agar plate were isolated and subjected to several alternating transfers on TCBS agar plates until pure colonies were established. Pure stock isolates were stored at -80 °C in a sterile balanced salt solution (BSS: 3.0% NaCl, 0.11% K_2_SO_4_, 0.135% NaH_2_PO_4_, 0.005% NaHCO_3_; pH 7.2) supplemented with 15% glycerol.

### Virulence test using a two-step immersion method

All of the isolated bacterial strains were separately inoculated at 30 °C for 24 h in marine broth 2216E (3% NaCl; Oxoid, USA) with constant shaking. The bacterial count was determined using standard dilution and plating methods as described by Hameed et al. [22]. Then, the strains were randomly divided into groups, with each group consisting of five strains. Apparently healthy 10 cm *P* *. andrewsi* nubbins were collected from natural sea areas in QLY as recipients in the following virulence test. To ascertain that the coral nubbins were disease-free prior to the experiment, they were acclimated in circulating filtered and UV-sterilized seawater for 10 d at 28 ± 2 °C in aquaria with complementary light in a roofed hatchery on Yongxin (Woody) Island. The experimental coral nubbins (test) were immersed overnight in 500 ml aerated SSW containing an inoculum of a mixture of five strains at a density of 10^5^ CFU ml^-1^. After inoculation, coral nubbins were transferred to aerated 10 l transparent plastic containers with lids and maintained at 28 °C to 30 °C, with five coral nubbins in each container. Three control groups (control), each also containing five nubbins, were inoculated in 500 ml aerated SSW with the same volume of marine broth 2216E. Water volumes in the test and control containers were adjusted periodically with sterile distilled water to maintain salinity. All nubbins in the test and control groups were checked daily for responses to the inoculations for a period of four weeks.

### Virulence test using a one-step immersion method

The experimental coral nubbins were collected and acclimated as described above. Each strain within the five-strain mixture group that resulted in obvious signs of disease in the previous two-step immersion inoculation was separately inoculated at 30 °C for 24 h in marine broth 2216E with constant shaking. The immersion method was similar to that of the two-step immersion, except that coral nubbins were immersed in SSW with an inoculum containing only one strain and maintained in the same blocked container during the entire period of four weeks. Thus, five treatments (each treatment representing one strain, five replicates in each treatment) and three controls (five replicates in each control) were performed for each mixed group of strains that produced a positive response in the two-step immersion. The inoculated coral nubbins were checked daily for a period of four weeks. Once disease signs were found on inoculated corals, six dominant colonies were re-isolated from diseased tissue with TCBS agar. The causality between an inoculated bacterial strain and tissue loss is established if the morphology of the dominant colony on TCBS agar was similar to that of the original strain used for inoculation. Except for the re-isolation, the virulence of the re-isolated colonies was also re-examined by one-step immersion as described above.

### Bacterial taxonomy

Part of the 16S rRNA gene of the virulent strain was amplified with the Unip1 (5ˊ-AGAGTTTGATCMTGGCTCAG-3ˊ, M = A/C/G, positions 8-27 in *Escherichia coli*) forward and Unip2 (5ˊ-GGTTACCTTGTTACGACTT-3ˊ, positions 1492-1510 in *E. coli*) reverse primers. PCR was performed with an annealing temperature of 58 °C for two minutes and an extension temperature of 72 °C for one minute. The amplified product was sequenced with an ABI Prism 3730 XL DNA Analyzer System (Applied Biosystems, Foster City, CA, USA). A Biolog GN III Microplate System (Biolog, Inc, Hayward, CA, USA), Bruker Biflex IV MALDI TOF spectrometer and MALDI Biotyper 2.0 software (Bruker Daltonics, Bremen, Germany) were triply used in parallel according to manufacturers’ recommendations. Additionally, classical phenotypes described by Noguerola and Blanch [23] and other taxonomic characteristics advised by Holt et al. [24] were used for classification, using *V* *. alginolyticus* ATCC33787 as a positive control. In brief, the selected strain was identified based on characteristics including Gram reaction (KOH method), cellular morphology, colony characteristics on TCBS agars, the production of arginine dihydrolase, lysine decarboxylase, ornithine decarboxylase, indole and gelatinase and growth at different temperatures (4, 30, 35 and 40 °C). Salt tolerance was estimated from growth in alkaline peptone water with 0%, 3%, 6%, 8% or 10% NaCl. Additionally, all of the re-isolations were characterized using the Biolog System.

## Results

### Transmission experiment in the field

The results are presented in Table 1. The response rate was defined as the number of responding replicates divided by the total number of replicates. No control replicates exhibited a disease response in either trial. Treatment replicates exhibited the same response rates (responding replicates/total replicates = 37.5%) during the first two weeks for both trials. By the end of the fourth week, although the response rate remained the same for the first trial, it increased to 50% for the second trial. Taken together, no replicates responded in the first five days; seven out of eight (87.5%) responding replicates exhibited symptoms within the first two weeks. In both trials, four out of eight diseased donors were covered by algae after the 5^th^ day. By the end of the fourth week, none of these algae-covered donors had caused any signs of disease in their corresponding test receptors. The difference in the number of replicates showing or not showing a disease response in the two trials was not statistically significant between test and control using Fisher’s exact test. However, the difference between test and control was statistically significant for trial 2 at 28 days if the number of effective replicates (excluding the invalid replicates where the donors were covered by algae after five days) is used to carry out Fisher’s exact test (Table 2).

**Table 1:** Results of the transmission experiment on *Porites* *andrewsi* .

Period	Treatment	---------------Trial 1---------------					----------------Trial 2----------------				
		n_1_	n_1_’	R_1_	P_1_	P_1_’	n_2_	n_2_’	R_2_	P_2_	P_2_’
14 days	Tested	8	4	3	0.2545	0.0714	8	4	3	0.2545	0.0714
	Control	4	4	0			4	4	0		
28 days	Tested	8	4	3	0.2545	0.0714	8	4	4	0.1414	0.0143
	Control	4	4	0			4	4	0		

n: number of replicates; n’: number of effective replicates, i.e., those that were tied to valid donors; R: number of replicates exhibiting a disease response; P: the value of the comparison between n and R using Fisher’s exact test; P’: the value of the comparison between n’ and R using Fisher’s exact test.

**Table 2:** Main phenotypic traits of strains XSBZ03 and XSBZ14 compared with *V* *. alginolyticus* .

Test methods	characteristics	Reactions		
		XSBZ03	XSBZ14	*V* *. alginolyticus* ATCC33787
Microscope	Gram stain	-	-	-
	Straight rod	+	+	+
Growth at	4°C	-	-	-
	30 °C	+	+	+
	35 °C	+	+	+
	40 °C	+	+	+
Growth on TCBS (30 °C, 24 h)	Color	Yellow	Yellow	Yellow
	Diameter of the colony	2~3 mm	2~4 mm	2~4 mm
	Swarming	+	+	+
Growth in alkaline peptone water with…	0% NaCl	-	-	-
	3% NaCl	+	+	+
	6% NaCl	+	+	+
	8% NaCl	+	+	+
	10% NaCl	+	+	+
Production of…	Arginine dihydrolase	-	-	-
	Lysine decarboxylase	+	+	+
	Ornithine decarboxylase	+	+	+
	Indole	+	+	+
	Gelatinase	+	+	+
Nitrate reduction		+	+	+
Voges–Proskauer reaction		+	+	+
Mannitol acid		-	-	-
ONPG*		+	-	-
Resistant to O/129 (10 µg ml^-1^)		+	+	+
Biolog GN III Microplate System (30°C, 24 h)	D-Melibiose	-	+	-
	β-Methyl-D Glucoside	+	-	-
	N-Acetyl-β-D Mannosamine^†^	-	+	+
	N-Acetyl Neuraminic Acid^†^	-	+	+
	D-Mannose	+	- (16) ^‡^	+
	3-Methyl Glucose^†^	-	+	+
	D-Serine	+	-	-
	D-Arabitol	- (5)^‡^	+	+
	*myo*-Inositol	-	+	-
	L-Histidine	- (23) ^‡^	+	+
	D-Glucuronic Acid	+	- (33) ^‡^	+
	Glucuronamide	+	-	-
	Quinic Acid	+	-	-
	D-Malic Acid	-	+	-
	γ-Aminobutryric Acid	+	-	-
	α-Hydroxybutyric Acid	+	-	-
	Propionic Acid	-	+	+
	Formic Acid^†^	-	+	+
	Inosine	+	+	-
	Rifamycin SV	+	+	-
	Methyl Pyruvate	+	+	-
	α-Keto-Glutaric Acid	+	+	-
	D-Malic Acid	- (10) ^‡^	- (8) ^‡^	+

* ONPG = ortho-nitrophenyl-β-D-galactopyranoside; †, the reactions are viable for different *V* *. alginolyticus* strains; ‡ the values in parentheses are less than the automatic threshold.

Rates of tissue loss ranged from 0.90 to 10.76 cm^2^ d^-1^ with a mean of 5.40 ± 3.34 cm^2^ d^-1^ (mean ± SD) in the two trials, which was much slower than what has been observed for natural transmission in the field (more than 30 cm^2^ d^-1^ in some cases). In all of the replicates that responded to the treatments, the signs first occurred at the site where the donor was in direct contact with the receptor, and then the signs extended along the branches (Figure 2). Based on the fact that on the 5^th^ day no diseased areas were observed yet on the 14^th^ day the diseased areas already exhibited a dull white color which indicates a past-infection status, the onset time of infection should have occurred at sometime between the 5^th^ and 14^th^ days, most likely around the 7^th^-10^th^ day. In all responding replicates, we observed that the closer the branches of the receptor coral were to the donor coral’s diseased areas, the bigger its diseased area was (Figure 2).

### Bacterial isolation

Suitable colonies on TCBS agar plates resulted from the 10^-1^ dilution for all six samples. All the diseased samples gave rise to three to five colony types (differing in color and size), and the most abundant colonies (> 65%) were yellow, with a diameter of 2-4 mm on the TCBS agar plate. For the healthy samples, five colony types were also observed, but no colony type was significantly more abundant than any other colony types. A total of 35 most abundant colonies were isolated from the TCBS agar plates of the three diseased samples and were numbered from XSBZ01 to XSBZ35.

### Virulence test

The 35 selected strains were randomly divided into seven groups, T_1_ -T_7_, in the two-step immersion virulence test. All inoculated nubbins appeared healthy in the first two days. However, the water of T_1_ became turbid after 48 h and the tissue of all five nubbins in T_1_ were lost and dead 3 d later, whereas the water remained clear and the nubbins were healthy for the other treatments and controls during the entire experimental period. The inoculum of T1 consisted of suspensions of the following five strains: XSBZ01, XSBZ03, XSBZ07, XSBZ14 and XSBZ22.

In the one-step immersion virulence tests, the *P* *. andrewsi* nubbins inoculated with strains XSBZ03 (10^5^ CFU ml^-1^) and XSBZ14 (10^5^ CFU ml^-1^) began to show disease signs on the 6^th^ and 9^th^ days of exposure, respectively. Once infected, in three to seven days, these nubbins became completely white and died. The coral nubbins in other treatments (n = 3) and the controls (n = 3) remained unaffected during the entire period of the experiment. The morphology of the dominant colony (more than 80%) isolated from the inoculated diseased nubbin was the same as that of the original strain used for inoculation. All of the twelve re-isolated colonies of strains XSBZ03 and XSBZ14 independently caused the same disease sign (tissue loss) on the coral after 6 d and 8 d in the one-step immersion, respectively (satisfying Koch’s postulates).

### Bacterial taxonomy

Nearly complete 16S rRNA gene sequences (1514 bp) for strains XSBZ03 (Genbank accession number: JX221044) and XSBZ14 (Genbank accession number: JX221045) were obtained. Both of them exhibited the highest similarity (99.8%) to the sequences of *V* *. alginolyticus* strain NBRC 15630. Additionally, the 16S rRNA gene of strain XSBZ03 had 99.8% identical nucleotides to that of *V. parahaemolyticus* strain CM12. The 16S rRNA gene of strain XSBZ14 also had 99.8% identical nucleotides to those of *V. natriegens* strain CM3 and *V. parahaemolyticus* strains RAMD 2210633 and CM12.

The results of the investigated biochemical reactions are listed in Table 2. Strains XSBZ03 and XSBZ14 were Gram-negative, straight rod, swarming, and positive for lysine decarboxylase and ornithine decarboxylase but negative for arginine dihydrolase. They were also positive for indole, gelatinase, nitrate reduction and Voges–Proskauer reactions and were slightly halophilic bacteria that required Na^+^ ions for growth and gave rise to yellow colonies on TCBS. Growth occurred at 40 °C but not at 4 °C for both strains. They were resistant to O/129 (10 µg ml^-1^). The mannitol acid reaction was negative for strains XSBZ03, XSBZ14 and *V* *. alginolyticus* ATCC33787, which was inconsistent with the result reported by Noguerola and Blanch [23]. Ortho-nitrophenyl-β-D-galactopyranoside (ONPG) was positive for strain XSBZ03 and negative for strain XSBZ14. Based on the reactions of 95 tested carbon sources in the Biolog GN III Microplate System, strains XSBZ03 and XSBZ14 had the highest similarities to *V* *. alginolyticus* (27.3%) and *V. splendidus* (19.0%), respectively. In the test of MALDI-TOF MS and MALDI Biotyper, the highest score values of strains XSBZ03 and XSBZ14 were 2.343 and 2.360, which were both beyond the threshold for secure identification at the level of species according to the manufacturers’ recommendations when compared with *V* *. alginolyticus* DSM 2171 HAM. The reactions of the six re-isolations of XSBZ03 or XSBZ14 were respectively as same as those of them in the Biolog System.

## Discussion

PAWS was widely observed during the field survey near the islands of the QLY in the South China Sea (2010 to 2011). Often, PAWS radiated outward from a central colony that was completely white. During the transmission experiment, tissue loss first appeared on areas of the receptor coral in direct contact with the white ("diseased") areas of the donor coral. Tissue loss subsequently extended along the receptor branch after the initial transmission occurred. However, once a coral was infected, PAWS was not confined to the same branch, other branches were also infected. We observed that the closer a branch was to the infection, the faster it showed signs of PAWS. Furthermore, the area of tissue loss was larger on the side that faced the branch of transmission. These features suggest that this disease is likely transmitted through water and could cause far-reaching and long-term epidemics. On the contrary, transmission was not successful if infected donor coral was covered by algae within the first five days of the experiment. This suggests that only the newly affected nubbin was infectious and that the pathogen was associated with the polyp or the symbiotic algae, but not the coral skeleton. The range and speed of tissue loss progression suggests that this disease may have recrudescent tendencies similar to white band disease in the Caribbean coral, *Acroporacervicornis* [10].

Considering the variability of reactions between different strains of *V* *. alginolyticus* and the possible false negative results due to the detection sensitivity limit of the Biolog system, the identification of strain XSBZ03 as *V* *. alginolyticus* seems to be most appropriate according to the highest similarity of 16S rRNA sequences between strain XSBZ03 and *V* *. alginolyticus* and the yellow colony on TCBS agar plate produced by strain XSBZ03. Previous studies showed that some *V* *. alginolyticus* strains might have almost identical 16S rRNA genes (even beyond 99.8% similarity) to *V. parahaemolyticus* [25]. Though strain XSBZ14’s 16S rRNA genes exhibited 99.8% similarity to those of *V. natriegens* and *V. parahaemolyticus*, it is distinctively different from *V. natriegens* and *V. parahaemolyticus* in several key characteristics such as swarming, indole and colony color on TCBS plates. Furthermore, all of the biochemical reactions and phenotypic traits of the two strains investigated in the study, with the exception of mannitol acid, were consistent with *V* *. alginolyticus* [23]. The identification of the two strains as *V* *. alginolyticus* was also supported by MALDI-TOF MS and MALDI Biotyper at the level of species according to the manufacturers’ recommendations. Thus, strains XSBZ03 and XSBZ14 were both identified as *V* *. alginolyticus* .

The results of this study showed that, on TCBS agar plates, the dominant colonies isolated from diseased samples were very different from those isolated from healthy samples. Simultaneously, two *V* *. alginolyticus* strains were proven to be the pathogenic agents for the disease of *P* *. andrewsi* . Although the exact mechanism of infection could not be pinpointed, the tissue loss of *P* *. andrewsi* was demonstrated to be an infectious epizootic disease caused by *V* *. alginolyticus* . Previously, *V* *. alginolyticus* has also been reported as the main cause of yellow band disease in Caribbean and Indo-Pacific reef-building corals [26] as well as many aquatic animals including fish, shrimps, shellfish and echinoids [12,27-30]. *V* *. alginolyticus* , a normal Gram-negative inhabitant of maricultural environments, has many genotypes, among which their virulence varies greatly [12,31]. Up to now, the molecular mechanism underlying the virulence of this pathogen is still unclear, and molecular diagnostics for the diseases caused by the bacterium have not been developed. Aiming to restore the destroyed coral ecosystem in the South China Sea, efforts to transplant *P* *. andrewsi* have been underway. Thus, to prevent this epidemic disease and ensure the successful implementation of *P* *. andrewsi* transplantation, it is urgent to further develop detection methods for the virulent strains of *V* *. alginolyticus* and robust diagnostics of the coral disease caused by *V* *. alginolyticus* in the South China Sea [32].

Coral holobionts associated with diverse assemblages of microorganisms make it difficult to isolate a single bacterial pathogen. Thus, the ability to rapidly determine specific pathogens would be the key to understanding coral epizootic diseases. In this study, to reduce the initial number of pathogenic candidates for the PAWS, a “shot-gun” method was employed. Briefly, before conducting a virulence test on each strain, the putative pathogenic strains were divided into five-strain groups and the virulence test was performed on a group basis first. After pin-pointing the most likely group to support the virulent strain, a second test was performed to determine whether virulence was detected in any member of that group. In order to obtain the balance between quickly isolating the highly virulent pathogen and avoiding the possible pathogenic synergy among the tested strains, two-step immersion was chosen for the group-based virulence test. In this way, the efficiency of accurate identification of the bacterial pathogens of coral disease was greatly improved. Our method may be useful for bacterial coral disease studies in the future.

In conclusion, due to the rapid tissue loss and unique pattern of infection, the PAWS epidemic that occurred in the South China Sea should not be easily confused with thermal coral reef bleaching [33-36]. We provide evidence to suggest that *V* *. alginolyticus* was the pathogenic agent responsible for this disease. These results also suggest that the disease is transferred through water and could cause far-reaching and prolonged infections. Several species of *Vibrio* are known marine pathogens, causing disease and death in corals as well as fish and shellfish [11-16]. We believe that the methods developed to identify culturable *Vibrio* spp. from corals will provide an effective and reproducible method for identifying other *Vibrio*-related marine pathogens in the future.
